# Supplementary material for: Evaluation of a Novel Prototype for Pressurized Intraperitoneal Aerosol Chemotherapy
Source: Cancers (Basel). 2020 Mar 9;12(3):633. doi: 10.3390/cancers12030633 (PMC7139407; doi:10.3390/cancers12030633)
Supplement: Supplementary file 1 [file cancers-12-00633-s001.zip › Table_S1.docx]

**Supplementary Materials**

| **Flow rate**  **(ml/min)** | **Height**  **(mm)** | **Mean particle velocity (m/s)** | **Mean diameter (μm)** | |
| --- | --- | --- | --- | --- |
|  |  |  | **Arithmetic mean diameter (μm)** | **Sauter mean diameter (μm)** |
| 30 | 120 | 1.31 | 25.4 | 32.1 |

**Table. S1.** Summary of the granulometric analysis of the nozzle prototype.
