# Supplementary figures and images for: Evaluation of a Novel Prototype for Pressurized Intraperitoneal Aerosol Chemotherapy
Source: Cancers (Basel). 2020 Mar 9;12(3):633. doi: 10.3390/cancers12030633 (PMC7139407; doi:10.3390/cancers12030633)

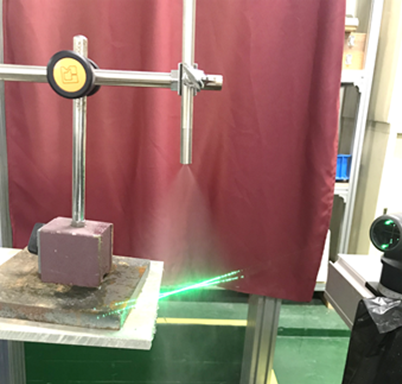

Supplement: Supplementary file 1 [file cancers-12-00633-s001.zip › Figure_S1.tif]
